# Towards Patient-Centered Care in Internal Medicine: A PLS-SEM Model of Shared Decision-Making and Patient Satisfaction Among Patients with Chronic Conditions

**DOI:** 10.3390/healthcare14203523

**Published:** 2026-10-19

**Authors:** Razvan Matei Bratu, Ionuț Bogdan Diaconescu, Ivona Răpan, Octavia-Sorina Honțaru, Cristina Niculescu-Ciocan, George Mihail Man

**Affiliations:** 1 Faculty of Medicine, University of Medicine and Pharmacy “Carol Davila” Bucharest, 050474 Bucharest, Romania; razvan.bratu@umfcd.ro; 2 Department of Anatomy, University of Medicine and Pharmacy “Carol Davila” Bucharest, 050474 Bucharest, Romania; 3 Institute of National Economy, Romanian Academy, 050711 Bucharest, Romania; stradivonas@gmail.com; 4 Faculty of Sciences, Physical Education and Informatics, Department of Public Health Argeș, Pitești University Center, National University of Science and Technology Politehnica Bucharest, 110040 Pitești, Romania; 5 Department for Teaching Staff Training, Faculty of Physical Education and Sports, National University of Physical Education and Sport in Bucharest, 060057 Bucharest, Romania; cristina1.niculescu@gmail.com; 6 Department of Medical Assistance and Physical Therapy, Faculty of Sciences, Physical Education and Informatics, University Center of Pitești, National University of Science and Technology Politehnica Bucharest, 110040 Pitești, Romania; george_mihail.man@upb.ro

**Keywords:** patient-centered care, shared decision-making, patient satisfaction, chronic conditions, internal medicine, doctor–patient communication

## Abstract

**Highlights:**

**What are the main findings?**

- Doctor–patient communication was positively associated with patient participation in medical decision-making.
- Patient participation in medical decision-making was strongly associated with patient satisfaction.

**What are the implications of the main findings?**

- Better communication can support patients in taking a more active role in medical decisions.
- Greater patient participation was associated with higher satisfaction with medical care.

**Abstract:**

**Background/Objectives**: Patient-centered care has become an important component of chronic disease management. Patients are increasingly involved in complex decisions about their treatment, while effective doctor–patient communication, access to clear medical information, and active involvement in decision-making can contribute to a better care experience. This study aimed to identify the main factors associated with patient participation in medical decision-making and to examine the relationship between participation and patient satisfaction. **Methods**: A cross-sectional study was conducted among 176 patients with chronic conditions. Data were collected using an online questionnaire. The proposed relationships between doctor–patient communication, trust in the doctor, ability to understand medical information, patient involvement in their own care, quality of information provided, participation in medical decision-making, and patient satisfaction were examined using partial least squares structural equation modeling (PLS-SEM). **Results**: All five factors examined were positively and significantly associated with patient participation in medical decision-making. Doctor–patient communication showed the strongest association, followed by patient involvement in their own care and ability to understand medical information. The model explained 69.3% of the variance in participation in medical decision-making (R^2^ = 0.693). Participation was strongly associated with patient satisfaction and explained 56.2% of its variance (R^2^ = 0.562). **Conclusions**: The findings highlight the importance of actively involving patients with chronic conditions in medical decision-making. Greater patient participation was associated with higher satisfaction with the care received.

## 1. Introduction

Chronic conditions create important challenges for healthcare systems, particularly as populations age and long-term care needs increase [1,2]. Technological developments are also changing healthcare delivery [3]. Patient-centered care places greater emphasis on patients’ needs, preferences, and experiences and is closely related to satisfaction and treatment adherence [4,5]. This approach reflects a shift from disease-centered to patient-centered care [6]. Person-centered models can support self-management and individualized care planning [7,8]. These approaches are particularly relevant to patients with multiple chronic conditions, who may experience fragmented care and require better coordination between healthcare professionals [9,10,11]. Shared decision-making allows patients and healthcare professionals to develop care plans that reflect patients’ goals and preferences [12]. Person-centered care planning can improve the care experience of patients with multiple chronic conditions [13]. However, patients may face difficulties in accessing coordinated care, while healthcare providers face organizational and resource-related challenges [9,14,15]. Shared decision-making is an important part of patient-centered care. It involves the exchange of information between patients and healthcare professionals and considers patients’ preferences when making medical decisions [16]. Digital tools may support access to medical information and care planning [17,18]. The need for individualized care is also relevant to patients with complex chronic conditions, including chronic liver disease [19].

### 1.1. Integrated Care, Patient Information and Continuity of Care

Patients with chronic liver disease may face complex care needs that require coordinated support [20]. Digital health services can help patients access care and medical information [21]. Communication between patients and healthcare professionals remains essential for person-centered care planning [22]. The integration of patient data across members of the care team and between primary care and behavioral health specialists is also crucial. By addressing both health and social risks through multidisciplinary teams, healthcare professionals can allocate time and resources more effectively in managing patients’ health [23]. In order to improve care for populations living with chronic illnesses or MCC, it is important to implement a strategic framework based on patient-centered care (PCC) and value-based healthcare (VBH). PCC emphasizes the importance of patients’ values and preferences in clinical decision-making. VBH seeks to provide high-quality healthcare at lower costs, while promoting timely, safe, and improved health outcomes [24]. Under optimal conditions, patients may benefit from lower costs and better health outcomes, while healthcare systems may achieve greater efficiency and improved delivery of care [25].

Integrated practice units (IPUs) consist of multidisciplinary teams that address specific medical conditions. These teams manage the full course of care, including supportive services such as nutrition, behavioral health, and social work. Mutual learning among healthcare professionals and better coordination of medical care may improve the performance of IPUs [26]. Patient-reported outcomes (PROs) can complement clinical indicators by providing a broader assessment of disease impact, diagnosis, and treatment effectiveness. PROs capture information regarding patients’ well-being, symptoms, physical and emotional functioning, and distress. They are increasingly viewed as direct indicators of high-quality patient-centered care. For example, the integration of PROs in oncology has been associated with improved patient–doctor communication [27,28]. During pre-visit planning, healthcare providers should improve the collection of PROs. These results should be available in real-time before clinical consultations, as they may simplify workflow and support healthcare professionals [29]. In addition, patient education regarding disease progression, complications, management, and preventive interventions remains essential. This can be supported through technological tools and educational resources made available through dedicated patient portals [30]. Patient- and system-level data, including blood tests, imaging, procedures, disease characteristics, and outcomes, are essential for developing an integrated data collection system [31]. Facilitating patient-centered and value-based care requires coordinated efforts at the patient, provider, system, and investment levels. At the patient level, this involves the acceptance of team-based care rather than exclusive reliance on the traditional doctor–patient model. At the provider level, it may involve taking on additional responsibilities. At the system level, procedures are needed to identify high-risk patients and provide appropriate ambulatory care. From an investment perspective, screening, preventive interventions, and long-term care should receive greater attention than episodic, high-cost treatment [32].

Among the challenges encountered by patients living with multiple chronic illnesses is the need for better integration of care, particularly between primary and hospital care. A lack of integration can result in repeated testing, fragmented care, and inefficient use of healthcare resources [33]. These organizational problems can negatively affect care quality, patient experience, and health outcomes, while also increasing the burden on healthcare professionals. A coordinated, long-term approach to chronic care based on multidisciplinary collaboration and shared decision-making is therefore essential [34]. Integration between medical specialties and different levels of care is important for ensuring comprehensive, effective, and continuous care. Better integration may facilitate earlier detection, reduce unnecessary procedures, and support more effective treatment and long-term monitoring of chronic conditions [35]. One example is the Spanish CARABELA project, launched in 2019. Its purpose is to transform the delivery of chronic care by placing continuity of care at the center of chronic disease management. The project also seeks to identify areas for improvement, propose scalable solutions, and define quality indicators. This methodology has been successfully applied to high-burden chronic illnesses such as severe asthma [36,37], heart failure [38], and chronic kidney disease [39]. Each of these CARABELA initiatives has contributed to the development of integrated, evidence-based, and adapted models of care aimed at improving service delivery [40]. In primary care, major priorities include digital tools, insufficient health education, and system overload, while in hospital care, the main priorities include professional training, protocol alignment, and continuity of care [41,42].

The implementation of shared electronic health records (EHRs) between primary and hospital care is important for strengthening coordination, improving care processes, and supporting the long-term management of chronic conditions [43,44]. Patients also require early diagnosis and a reduction in delayed diagnosis, particularly in cases involving acute episodes [45]. Patient education is a core component of chronic disease management and plays an important role in improving understanding, self-monitoring, and the early detection of symptoms [46]. Healthcare professionals working in internal medicine need appropriate training to improve the detection and management of chronic illnesses. They should also be able to provide patients with clear and detailed information regarding the disease and its management, particularly at the time of diagnosis. This can be supplemented by written or digital information designed to reinforce medical guidance [47]. Important topics include the impact of the condition on quality of life and the ways in which physical activity, diet, work, social life, and travel may be affected. Therefore, collaboration and communication between different levels of care become particularly important [48,49].

### 1.2. Patient-Centered Care and Shared Decision-Making in Internal Medicine

Patient-centered care (PCC) is increasingly regarded as a central component of internal medicine because it recognizes patients as active partners in their own care. Through this approach, their individual needs, values, and preferences are taken into account. The main objectives of PCC include improving health outcomes, increasing patient satisfaction, and providing high-quality care through shared decision-making, strong patient–provider relationships, and individualized care plans [50]. The importance of patient centricity has also been highlighted in empirical healthcare research examining patients’ perceptions and experiences with medical care [51]. Within internal medicine, the implementation of patient-centered care has the potential to address complex health needs and support preventive care. One of the approaches that incorporates the principles of PCC is shared decision-making. This is an important strategy based on collaboration between healthcare professionals and patients when treatment choices are made. Such collaboration may contribute to better-informed and more satisfied patients, as well as improved adherence to care plans. Shared decision-making (SDM) focuses on collaboration between patients and healthcare professionals during treatment-related decisions. In this process, patients’ preferences are taken into consideration, which may contribute to higher levels of satisfaction [52]. Through SDM, patients can better understand the available healthcare options and take greater control over their care, potentially leading to better treatment adherence than in conventional care models [53]. Nevertheless, the implementation of PCC models in internal medicine may encounter several challenges, including limited resources, increased workload, and inconsistencies in professional training. Successful implementation requires team collaboration, organizational changes, and supportive policies [54]. Healthcare providers also face high workloads, time pressure, and the need for additional training. These challenges may be addressed through adequate organizational support and appropriate resources [55]. Successfully implemented PCC models may also provide benefits for healthcare professionals by promoting a collaborative environment rather than a traditional hierarchical model of healthcare delivery [56]. PCC can contribute to improvements in safety, efficiency, public health, and patient satisfaction. Other potential benefits include better healthcare quality and efficiency, particularly in internal medicine, where patients often require comprehensive and continuous care [57]. Healthcare professionals caring for patients with chronic diseases have shown increasing interest in providing patient-centered care. Greater attention is being given to individual patient journeys based on interactions occurring before, during, and after the provision of healthcare services [58].

### 1.3. Conceptual Framework

Previous studies have shown that patient-centered care, patient involvement, patient participation in medical decision-making, and shared decision-making are closely related concepts. However, they represent different aspects of healthcare delivery. Patient-centered care refers to the organization and delivery of healthcare services according to the needs and preferences of each patient. Patient involvement refers to the active role of patients in managing their own health. It also includes following medical recommendations, monitoring their health, and taking responsibility for certain aspects of their care. Patient participation in medical decision-making refers to the extent to which patients are involved in decisions about medical investigations, treatment, and care management. Shared decision-making is a collaborative process. During this process, healthcare professionals and patients exchange information, discuss available options, and consider patients’ preferences when making medical decisions. Therefore, patient participation should be considered an important component of shared decision-making.

The conceptual model proposed in this study aims to illustrate these differences. Doctor–patient communication and the quality of medical information can facilitate patient participation in medical decisions. Trust in the doctor is another factor that may encourage patients to express their needs and preferences. The ability to understand and use medical information reflects patients’ perceptions of their own ability to interpret the information they receive. Involvement in their own care refers to the extent to which patients actively participate in managing their chronic conditions. All the variables included in the conceptual model are conceptually distinct. However, each of them may play an important role in patient participation in medical decision-making. As shown in the proposed model, patient participation in medical decision-making occupies an intermediate position. It represents a specific aspect of the healthcare experience through which communication, trust, information-related factors, and broader patient involvement may be associated with satisfaction. When patients feel that they have the opportunity to participate in decisions about their care, their healthcare experience may become more positive. They may feel that their needs and preferences are being heard and taken into account. This may also be associated with a higher level of satisfaction with the care received. Based on these considerations, the proposed model examines the relationships between five dimensions of patient-centered care and patient participation in medical decision-making. In addition, the study examines the relationship between patient participation in medical decision-making and the level of satisfaction reported by patients.

Based on the theoretical considerations presented above, a quantitative study was conducted among patients with chronic conditions who had received internal medicine services. The study aimed to identify the main factors associated with patient participation in medical decision-making. It also examined the relationship between patient participation and satisfaction with the medical care received. For this purpose, a conceptual model was developed. The model included seven variables: doctor–patient communication, trust in the doctor, ability to understand medical information, patient involvement in their own care, quality of information provided by the doctor, patient participation in medical decision-making, and patient satisfaction. The first five constructs were measured using multiple statements, while patient participation in medical decision-making (PPMD) and patient satisfaction (PS) were each measured using a single item.

### 1.4. Research Hypotheses

The hypotheses formulated within the model were based on a number of previous studies identified in the literature. The research hypotheses were as follows:

**Hypothesis** **H1.** *The quality of doctor–patient communication is positively associated with patient participation in medical decision-making.*

Communication between doctor and patient is considered one of the most important components of patient-centered care. The communication process between the two parties facilitates the exchange of information and patient involvement in the decision-making process. A study conducted in the literature [59] showed that certain forms of communication can encourage patients to become more active in decision-making. Siebinga et al. [60] showed the existence of a relationship between patient-centered communication and shared decision-making, although the two concepts remain distinct. Other studies have also shown that communication and information exchange are two essential elements of the decision-making process [61]. On the other hand, Tian et al. [62] showed that better communication skills among doctors lead to improved satisfaction with involvement.

**Hypothesis** **H2.** *Patients’ trust in the doctor is positively associated with patient participation in medical decision-making.*

Trust is considered an important component of the relationship between the doctor and patient. It influences the way patients position themselves in their relationship with the doctor, as well as their involvement in the decision-making process. Kraetschmer et al. [63] showed the existence of a relationship between patients’ trust in their doctor and their involvement in decision-making. Thus, shared decision-making was found to be associated with a certain level of trust in the doctor. Tian et al. [62] also showed that there is a relationship between trust and the degree of patient participation in decision-making. Moreover, the study showed that trust also influences the level of satisfaction experienced by patients. Strokes et al. [64] also illustrated the existence of a close relationship between trust, the communication process, and shared decision-making.

**Hypothesis** **H3.** *Patients’ ability to understand and use medical information is positively associated with patient participation in medical decision-making.*

The ability of patients to understand and use medical information is very important for their active participation in the decision-making process. Stacey et al. [65] considered health literacy an important condition for understanding information. Moreover, it contributes substantially to health-related decision-making. Shen et al. [66] showed in their study that there is a relationship between health literacy and shared decision-making. On the other hand, Holden et al. [67] showed that a higher level of health literacy leads to more active patient participation. Richter et al. [68] noted that in order to facilitate the shared decision-making process, communication about the benefits and risks needs to be adapted to the patient’s level of health literacy.

**Hypothesis** **H4.** *Patient involvement in their own care is positively associated with patient participation in medical decision-making.*

Smith et al. [69] showed in their research that a higher level of patient activation leads to a more favorable perception of the benefits of shared decision-making. On the other hand, Poon et al. [70] conducted a study among patients with diabetes and/or cardiovascular disease and examined the relationship between patient activation and the shared decision-making experience. The results showed a direct and positive relationship between the two variables.

**Hypothesis** **H5.** *The quality of information provided by the doctor is positively associated with patient participation in medical decision-making.*

Access to relevant and easy-to-understand information is essential for patient participation in decision-making. Previous research among patients with chronic conditions has also considered the information provided by the doctor as an important factor associated with patients’ medical decisions [71]. Bomhof-Roordink et al. [61] highlighted the importance of presenting treatment options to patients for their participation in medical decision-making. Sepucha et al. [72] identified information quality and communication with patients as important variables that directly influence the quality of complex and shared decision-making processes. Other authors who studied this field [73] highlighted the importance of educational materials and tools designed for patients in facilitating their involvement in the decision-making process. On the other hand, Richter et al. [68] showed that the way information about the benefits and risks of different options is communicated can directly influence shared decision-making.

**Hypothesis** **H6.** *Patient participation in medical decision-making is positively associated with patient satisfaction.*

The relationship between shared decision-making and patient satisfaction has been studied by many researchers in the field. Joosten et al. [74] examined the relationship between shared decision-making (SDM), satisfaction, adherence, and health status. They showed that SDM can produce favorable effects in the case of chronic diseases. Other authors [75] showed that patient involvement in the decision-making process positively influences patient satisfaction. On the other hand, Shay and Lafata [76] showed that SDM strongly influences satisfaction in general, while Sainfort and Booske [77] identified a positive association between shared decision-making and satisfaction with the medical decision. Siebinga et al. [60] showed that a high level of SDM, together with a patient-centered communication process, can lead to increased patient satisfaction.

**Figure 1:**
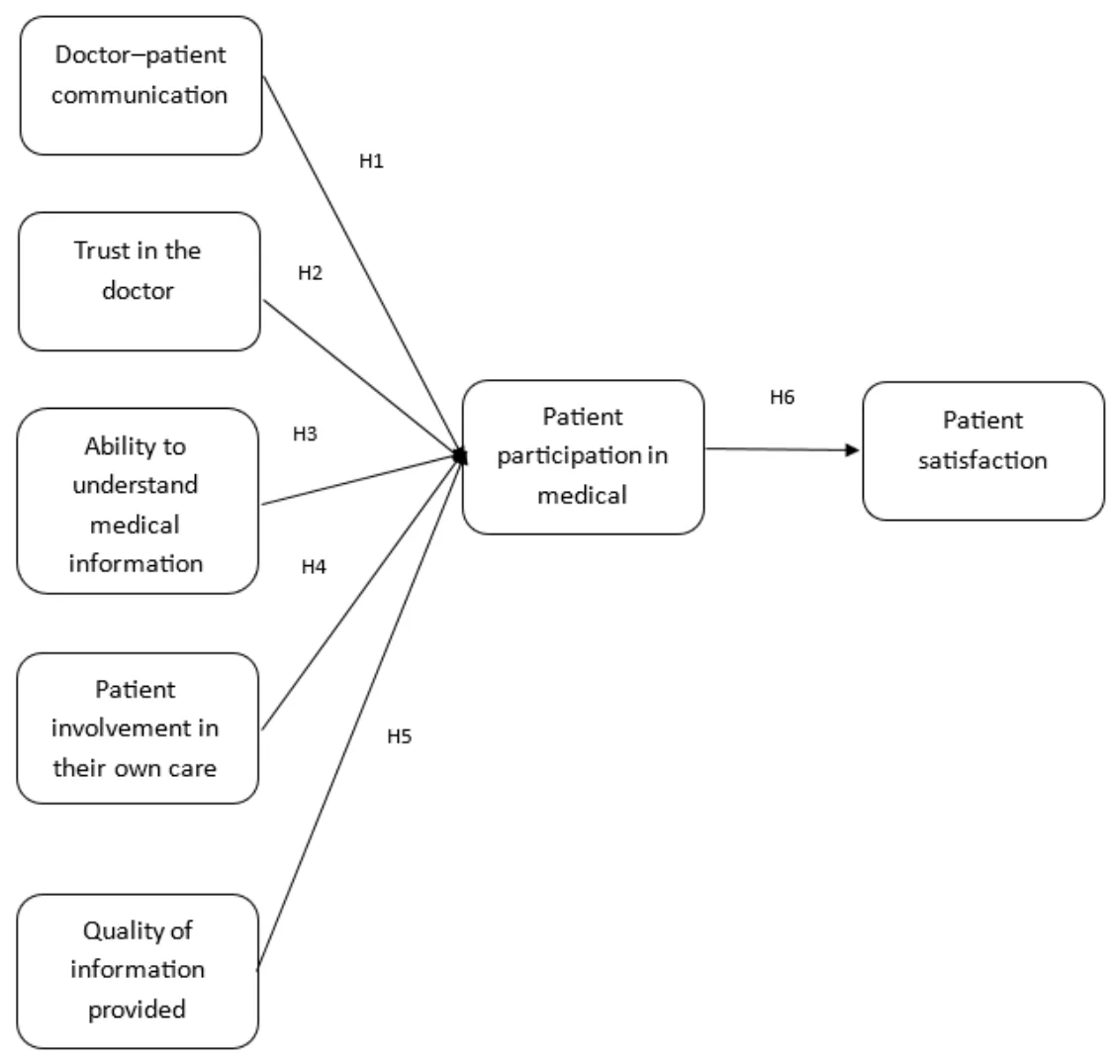
Proposed conceptual model of patient participation in medical decision-making and patient satisfaction in internal medicine.

Figure 1 below illustrates the proposed conceptual model of patient participation in medical decision-making and patient satisfaction in internal medicine.

## 2. Materials and Methods

The research was conducted between June and September 2025. The study was carried out on a sample of 176 respondents. The aim of the research was to identify the main factors associated with patient participation in medical decision-making. The study also examined the relationship between patient participation in medical decision-making and patient satisfaction. The quantitative study was conducted among patients with chronic conditions who had received internal medicine services in Romania in the previous months. Data were collected using a questionnaire. The research instrument was developed in an online format and subsequently distributed to the respondents. It was designed based on the objectives of the study and the hypotheses presented above. The proposed model aims to examine the relationships between doctor–patient communication, trust in the doctor, the patient’s ability to understand and use medical information, patient involvement in their own care, and the quality of the information provided. These variables were analyzed in relation to patient participation in medical decision-making. In the final part of the model, the relationship between participation in medical decision-making and patient satisfaction was analyzed. Multiple statements were used to measure the first five constructs, whereas PPMD and PS were each measured using a single item. Similar quantitative approaches based on structural equation modeling have been used in previous research to examine relationships between latent constructs and satisfaction-related outcomes [78]. The sampling method used was snowball sampling. Before the final distribution of the questionnaire to patients, a pretesting stage was conducted. Its purpose was to identify possible wording or comprehension problems. The questionnaire was pretested on a sample of 30 patients. After the pretesting stage and the identification of the existing problems, several changes were made to the questionnaire, after which it was distributed to the respondents.

Partial least squares structural equation modeling (PLS-SEM) was used to examine the proposed relationships between the study variables. The main objective was to identify the factors associated with patient participation in medical decision-making and to examine its relationship with patient satisfaction. PLS-SEM was considered suitable because it allowed several relationships to be examined within the same model. It also allowed the measurement properties of the multi-item constructs to be assessed. Unlike conventional regression, PLS-SEM allowed us to examine the measurement model and the structural relationships within a single analysis. Covariance-based SEM would also have been a possible approach. PLS-SEM was selected because the study focused on explaining variation in patient participation and satisfaction rather than assessing overall model fit. Given the relatively simple structural model, conventional regression could also be considered in future research.

The model included five constructs measured with four items each and two variables measured with a single item. The analysis was conducted in WarpPLS 8.0 using data from 176 patients. PLS Regression was used for the measurement model, while the Warp3 algorithm was applied to the structural model. Statistical significance was assessed using bootstrapping with 999 resamples. The resulting standard errors were used to calculate approximate 95% confidence intervals for the path coefficients. Sample-size adequacy was also examined using a sensitivity power analysis for multiple regression. The analysis considered five predictors, 176 participants, and a significance level of 0.05. With 80% statistical power, the sample could detect an overall effect size of approximately f^2^ = 0.075. This calculation provides an indication of sample-size adequacy for the main regression equation. It does not assess the power of each individual path in the PLS-SEM model.

To ensure that only respondents belonging to the target population participated in the study, the first question of the questionnaire was used as a screening question. Only individuals who, during the previous 12 months, had been consulted, evaluated, or treated in an internal medicine office, outpatient clinic, or internal medicine department for a chronic condition were able to continue the questionnaire. The first part of the questionnaire then included several questions regarding the patients’ medical profile. These covered the main chronic condition for which they had received care, the time since diagnosis, the type of healthcare facility where the most recent consultation took place, and whether they were currently receiving regular treatment. In addition, respondents were asked whether a decision regarding investigations, treatment, or medical management had been made during the consultation. Another important part of the questionnaire included the variables of the conceptual model. These sections examined doctor–patient communication, trust in the doctor, the ability to understand and use medical information, patient involvement in their own care, the quality of the information provided, patient participation in medical decision-making, and patient satisfaction. The relationships between the variables were measured using a seven-point Likert scale. In addition to the variables included in the model, the questionnaire contained several questions concerning possible improvements to internal medicine services.

### Questionnaire Design

The questionnaire used in this study was designed to collect information from patients with chronic conditions who had received internal medicine services. Its development took into account both the research objectives and the hypotheses established within the conceptual model. The questionnaire consisted of 22 questions, divided into several sections according to the objectives of the study. As above-mentioned, only patients with chronic conditions who had received internal medicine services during the previous 12 months participated in the study. The questionnaire was divided into the following sections:

Respondent screening section—The first question was used to verify whether respondents belonged to the target population. Individuals who did not meet the criterion established by the screening question were not able to complete the questionnaire.

- Section 1—Patients’ medical profile
  ○ This section included questions Q2–Q6. Information was collected regarding the main chronic condition for which patients had received care, the duration of the disease, and the type of healthcare facility where their most recent consultation took place. Respondents were asked to indicate only the main chronic condition for which they had received internal medicine care. In addition, respondents were asked whether they were currently receiving regular treatment and whether a decision related to investigations, treatment, or medical management had been made during the consultation.
- Questionnaire Part 2—Patient–doctor relationship and communication
  ○ Questions Q7 and Q8 focused on two aspects. The first concerned communication between the doctor and the patient, while the second concerned trust in the doctor. The main aspects examined included the clarity of explanations, the attention given to the patient, the opportunity to ask questions, the respectful nature of the dialogue, trust in the doctor’s competence, and the sense of safety associated with the doctor’s recommendations.
- Questionnaire Part 3—Patient ability to understand and manage medical information
  ○ This section included questions Q9 and Q10. Question Q9 examined the patients’ ability to understand and use the medical information they received. The following question analyzed patient involvement in their own care. The aspects considered included understanding their health condition, the role of treatment, the use of medical information, adherence to recommendations, and monitoring their own health.
- Questionnaire Part 4—Quality of information and participation in medical decision-making
  ○ Questions Q11 and Q12 were used to collect information on two central elements of the proposed model. The first examined the quality of the information provided by the doctor, while the second focused on patient participation in medical decision-making.
- Questionnaire Part 5—Patient satisfaction
  ○ Question Q13 was designed to measure overall patient satisfaction. Patients were asked to evaluate their overall satisfaction with the internal medicine services received.
- Questionnaire Part 6—Aspects that could be improved in the future
  ○ This section included questions Q14–Q18. These questions focused on patients’ perceptions of aspects that could be improved in internal medicine services. The time allocated to the consultation, clarity of explanations, communication, involvement in decision-making, and access to investigations were analyzed. Patients were also asked how much they would like to be involved in decisions regarding their treatment and what forms of support could help them participate more actively in their own care.
- Questionnaire Part 7—Demographic profile of respondents
  ○ The final part of the questionnaire consisted of questions Q19–Q22. These questions collected information regarding the respondents’ sex, age, level of education, and area of residence.

A seven-point Likert scale (1—“Strongly disagree” to 7—“Strongly agree”) was used to measure the variables included in the conceptual model. The scale was used only for the questions corresponding to the variables included in the model. The variables doctor–patient communication (DPC), trust in the doctor (TD), ability to understand medical information (AUMI), patient involvement in their own care (PIC), and quality of information provided (QIP) were measured using four statements. Therefore, the analysis was conducted using a total of 20 items. Patient participation in medical decision-making (PPMD) and patient satisfaction (PS) was each measured using a single item. This approach was chosen to obtain a direct assessment of the patients’ experiences. The PPMD item focused on whether patients perceived that medical decisions were made together with their doctor. The PS item measured overall satisfaction with the internal medicine care received. Both items were designed to capture a global perception rather than the different dimensions of these concepts. This approach also helped keep the questionnaire concise for patients with chronic conditions. However, single-item measures provide a narrower assessment of these constructs than multi-item scales.

The questionnaire was developed specifically for this study. Its development was based on the research objectives and on previous studies identified in the literature. These studies helped us select the main variables included in the conceptual model. The questionnaire items were then formulated to examine these variables in the context of internal medicine services provided to patients with chronic conditions. The existing literature also supported the proposed relationships between the variables [59,60,61,62,63,64,65,66,67,68,69,70,71,72,73,74,75,76,77]. Doctor–patient communication (DPC) was measured using four items. These examined whether the doctor explained health problems in a way that patients could understand, listened carefully, encouraged questions, and communicated clearly and respectfully. Previous research has highlighted the importance of communication in patient participation in medical decisions [59,60,61,62]. Trust in the doctor (TD) was also measured using four items. These focused on patients’ trust in the doctor’s professional competence, confidence in the recommended treatment, belief that the doctor acted in their best interest, and feelings of safety when following medical recommendations. The importance of trust in the doctor–patient relationship has also been examined in previous studies [62,63,64]. The ability to understand and use medical information (AUMI) included four items. These examined patients’ understanding of their health condition, the role of treatment in managing their condition, the medical terms used by the doctor, and their ability to use the information received when making decisions about their health. Previous studies have examined the relationship between health literacy and patient participation in medical decision-making [65,66,67,68]. Patient involvement in their own care (PIC) was measured using four items. These focused on active involvement in managing a chronic condition, following medical recommendations, monitoring one’s health when necessary, and recognizing one’s own role in managing health. The importance of patient involvement has also been discussed in previous research [69,70]. The quality of information provided (QIP) was measured using four items. These examined whether patients received sufficient information about their condition, whether the information was clear and easy to understand, whether treatment options were explained, and whether the information was useful for managing their health. The role of information quality in medical decision-making has been highlighted in previous studies [61,68,71,72,73].

Patient participation in medical decision-making (PPMD) was measured using a single item. It examined whether patients perceived that decisions about their health were made together with their doctor. Previous research has addressed the importance of patient participation and shared decision-making [59,60,61,62,63,64,65,66,67,68,69,70,71,72,73,74,75,76]. Finally, patient satisfaction (PS) was measured using a single item. It examined patients’ overall satisfaction with their experience of receiving internal medicine care. Previous studies have also examined the relationship between shared decision-making and patient satisfaction [60,74,75,76,77]. A seven-point Likert scale was used to measure all seven variables, ranging from 1 (strongly disagree) to 7 (strongly agree).

In the proposed model, the first five variables were analyzed as factors associated with patient participation in medical decision-making. Participation in decision-making was subsequently analyzed in relation to patient satisfaction. Questions Q14–Q22 were included to complement the analysis conducted within the conceptual model. They provide relevant information regarding patient expectations, aspects that could be improved, and the demographic characteristics of the sample.

## 3. Results

### 3.1. Respondents’ Profile

Regarding the respondents’ profile, all participants in the study reported that they had been evaluated or treated for a chronic condition within the previous 12 months. Regarding the main chronic condition reported by respondents, hypertension was the most frequently reported condition (33.5%), followed by cardiovascular diseases (17.6%) and diabetes mellitus (17.0%). Metabolic or endocrine disorders were reported by 9.7% of respondents, digestive or liver conditions by 9.1%, chronic respiratory diseases by 8.0%, and chronic kidney conditions by 5.1%. Respondents were classified according to the main chronic condition reported; therefore, these categories were mutually exclusive. Regarding the duration of the disease, 36.4% of participants reported having had their condition for more than 10 years, while 27.3% had been living with it for 6–10 years. For 15.3% of respondents, the duration was 4–5 years, for 14.2% it was 1–3 years, and for 6.8% it was less than one year. Regarding their most recent consultation, most respondents reported that it took place in the outpatient department of a public hospital (38.6%). A further 29% reported having been admitted to a public hospital, 24.4% had a consultation at a private clinic or medical practice, and 8% had been admitted to a private healthcare facility. Most participants (85.8%) reported receiving regular treatment for one or more chronic conditions, while 14.2% were not receiving such treatment. Among the patients who participated in the study, 89.2% reported that they had been involved in making a decision regarding investigations, treatment, or medical management during their consultation or episode of care. The remaining 10.8% reported that they had not been involved in such a process. Regarding the demographic profile of the respondents, 56.8% were women and 43.2% were men. In terms of age distribution, 32.4% were over 65 years old, followed by those aged 56–65 years (25.6%) and those aged 46–55 years (19.9%). Respondents aged 36–45 years accounted for 11.4% of the sample, those aged 26–35 years for 7.4%, and those aged 18–25 years for 3.4%. Regarding the educational level of the patients who participated in the study, most had bachelor’s degree (60.8%), followed by those with a master’s degree (23.9%), a high school education (12.5%), and a doctoral degree or MBA (2.8%). In terms of place of residence, 65.9% of respondents lived in urban areas, while 34.1% lived in rural areas.

### 3.2. Patients’ Views on Potential Improvements in Internal Medicine Care

Regarding the aspects of medical care that should be improved, the main aspect mentioned by respondents was access to medical investigations and healthcare services (31.8%), followed by the time allocated to consultations (24.4%) and the information provided about treatment and available alternatives (14.2%). Among the participants, 11.4% mentioned that communication and the attitude of medical staff should be improved, 9.7% indicated the clarity of the explanations provided by the doctor, and 8.5% mentioned patient involvement in decision-making. Regarding the respondents’ desire to be more involved in decisions about their treatment or medical care, 39.2% of participants reported that they would like to be involved to a great extent, while 23.9% stated that they would like this to a very great extent and 23.9% to a moderate extent. Only 8.5% would like to be more involved to a small extent, while 4.5% did not want greater involvement at all. For more active participation in their own care, 28.4% of participants mentioned the need for easier communication with the doctor between consultations, while 25.6% indicated the need for more time to ask questions. A further 23.9% of respondents mentioned the need for more detailed explanations from the doctor. On the other hand, 14.2% of patients reported that access to reliable online medical information would be useful, while 8% mentioned the need for greater involvement of family members or caregivers. Finally, 83.5% of participants stated that they would like their doctor to present more alternatives when several treatment options are available, while 16.5% did not express this preference.

### 3.3. Reliability Analysis

The reliability of the constructs was assessed using Cronbach’s alpha and composite reliability. The results showed high internal consistency for all multi-item constructs. Cronbach’s alpha values ranged from 0.829 to 0.860. Composite reliability values ranged from 0.887 to 0.905. All values were above the recommended threshold of 0.70. Convergent validity was assessed using the average variance extracted (AVE). The values ranged from 0.662 for AUMI to 0.704 for DPC. Therefore, all AVE values were above the recommended level of 0.50. These results indicate adequate convergent validity for the five multi-item constructs. PPMD and PS were measured using single items and were not assessed for internal consistency in the same way as the other constructs (Table 1).

The inner VIF values were examined to identify possible collinearity among the predictor constructs. For PPMD, the values ranged from 1.058 to 1.146. The VIF value for PPMD as the only predictor of PS was 1.000. All values were below the threshold of 3.3. Therefore, no relevant collinearity problems were identified in the structural model (Table 2).

Discriminant validity was assessed using the Fornell–Larcker criterion. For each multi-item construct, the square root of the AVE was compared with its correlations with the other constructs. The diagonal values ranged from 0.814 to 0.839 for the five multi-item constructs. In each case, these values were higher than the corresponding correlations with the other constructs, supporting discriminant validity according to the Fornell–Larcker criterion. PPMD and PS were measured using single items; therefore, their diagonal values of 1.000 were considered separately from those of the multi-item constructs (Table 3).

Discriminant validity was also assessed using the heterotrait–monotrait ratio (HTMT). This analysis was performed for the five constructs measured using multiple items. All HTMT values were below the threshold of 0.85, ranging from 0.118 to 0.317 (Table 4). The values were also low for doctor–patient communication, quality of information provided, and ability to understand medical information. These results provide further support for the discriminant validity of the multi-item constructs.

KMO and Bartlett’s test were calculated as preliminary assessments of the suitability of the 20 multi-item indicators for factor analysis. The Kaiser–Meyer–Olkin (KMO) measure was 0.778, indicating adequate sampling adequacy for factor analysis. Bartlett’s test of sphericity was significant (χ^2^ = 1603.449, df = 190, *p* < 0.001). This result shows that the correlations among the items were adequate for factor analysis (Table 5).

### 3.4. PLS-SEM Structural Model and Hypothesis Testing

**Figure 2:**
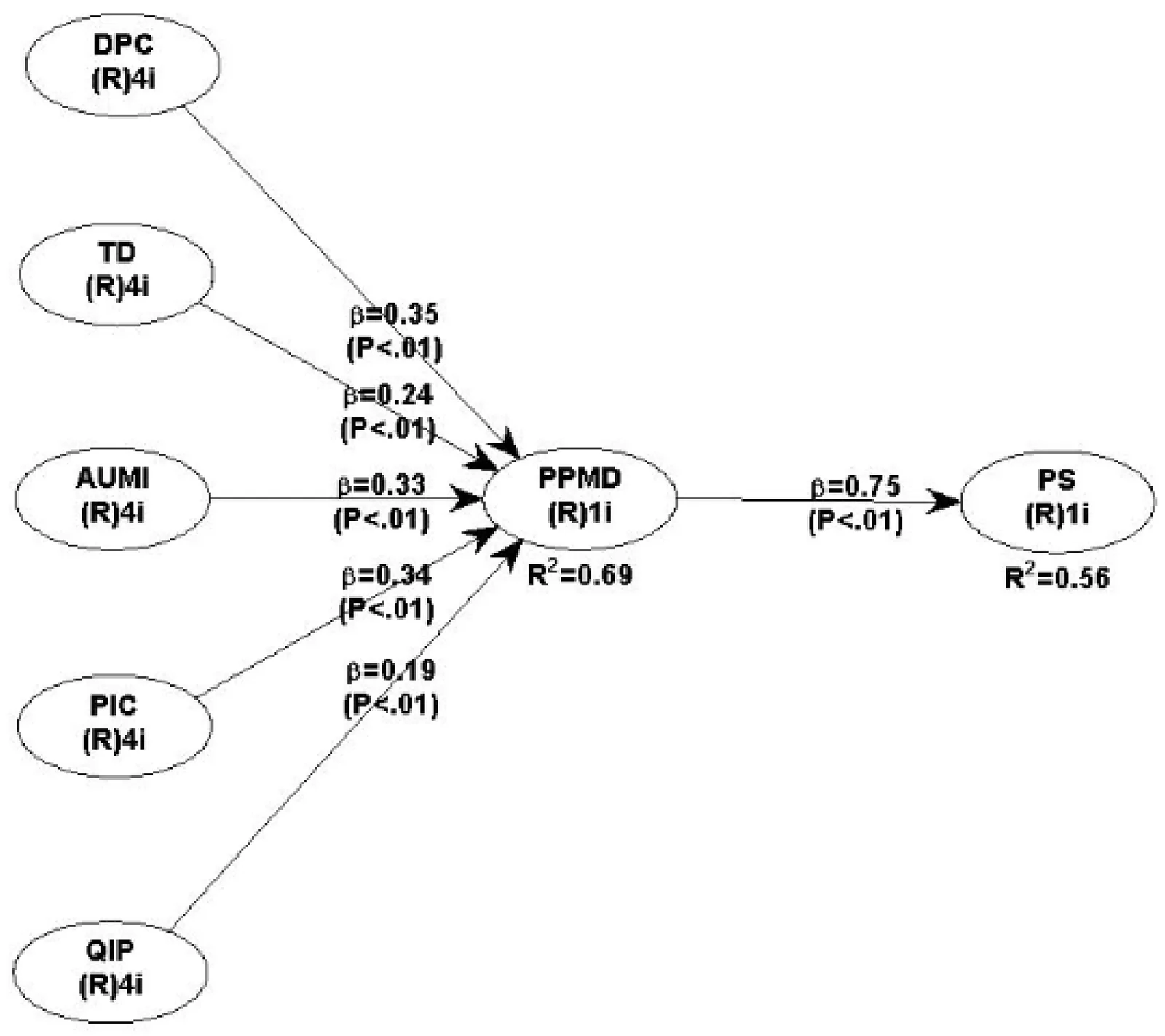
Structural model tested using WarpPLS 8.0.

The proposed structural model was tested using WarpPLS 8.0. The analysis examined the factors associated with patient participation in medical decision-making (PPMD) and the relationship between PPMD and patient satisfaction (PS). Five predictors of PPMD were included in the model: doctor–patient communication (DPC), trust in the doctor (TD), ability to understand medical information (AUMI), patient involvement in their own care (PIC), and quality of information provided (QIP). Six hypotheses were tested. The results showed that all five predictors had positive and statistically significant relationships with PPMD (Figure 2).

The results showed that doctor–patient communication was positively associated with patient participation in medical decision-making (β = 0.35, *p* < 0.01), supporting H1. Trust in the doctor also showed a positive relationship with PPMD (β = 0.24, *p* < 0.01). Therefore, H2 was supported. A positive association was also found between the ability to understand medical information and PPMD (β = 0.33, *p* < 0.01), providing support for H3. Patient involvement in their own care was positively related to participation in medical decision-making (β = 0.34, *p* < 0.01), supporting H4. The quality of information provided also had a positive and significant relationship with PPMD (β = 0.19, *p* < 0.01). Thus, H5 was supported. The final hypothesis examined the relationship between patient participation in medical decision-making and patient satisfaction. A strong positive and statistically significant relationship was found between the two variables (β = 0.75, *p* < 0.01). Therefore, H6 was supported. This was also the strongest relationship observed in the structural model. The result indicates that greater participation in medical decision-making was associated with higher patient satisfaction. The effect sizes ranged from 0.086 to 0.562. The highest value was found for the relationship between patient participation in medical decision-making and patient satisfaction (0.562). The lowest value was found for the relationship between quality of information provided and patient participation (0.086) (Table 6).

The approximate 95% confidence intervals did not include zero for any of the six relationships examined. These results support the positive associations identified in the structural model.

The explanatory power of the model was assessed using the coefficient of determination (R^2^). The R^2^ value for PPMD was 0.69. This means that the five predictors included in the model explained approximately 69.3% of the variance in patient participation in medical decision-making. For patient satisfaction, the R^2^ value was 0.56. Thus, approximately 56.2% of the variance in patient satisfaction was explained by patient participation in medical decision-making. Predictive relevance was assessed using the Q^2^ coefficients provided by WarpPLS 8.0. The Q^2^ values were 0.696 for patient participation in medical decision-making and 0.563 for patient satisfaction. Both values were above zero, indicating predictive relevance for the two endogenous variables (Table 7).

The model fit and quality indices indicated an adequate overall fit of the structural model. The average path coefficient (APC = 0.366, *p* < 0.001), average R-squared (ARS = 0.627, *p* < 0.001), and average adjusted R-squared (AARS = 0.621, *p* < 0.001) were statistically significant. The AVIF (1.089) and AFVIF (1.954) values were below the recommended threshold of 3.3. These values indicate no relevant collinearity problems. The Tenenhaus goodness-of-fit (GoF) index was 0.699 (Table 8). According to the conventional thresholds reported in the WarpPLS output, this value falls within the large GoF category. However, the GoF index was interpreted descriptively and was not considered, on its own, evidence of overall model fit. In addition, SPR, RSCR, SSR, and NLBCDR were all equal to 1.000 and met their recommended criteria. Overall, these results indicate that the proposed structural model shows satisfactory fit and quality.

## 4. Discussion

Patient-centered care can be achieved by involving and informing patients and by taking their perspectives into account. Patient engagement shifts the focus from the disease alone toward the broader well-being of the individual. PCC has been examined in different clinical contexts, including psychological conditions and cardiovascular risk [79]. At the same time, the increasing integration of artificial intelligence into clinical practice highlights the importance of understanding how healthcare professionals perceive and intend to use emerging technologies in patient care [80]. A key objective of PCC is to involve patients in decisions about their care. This is particularly important for patients with multiple chronic conditions, as treatment decisions may involve different benefits, risks, and individual preferences [81]. Communication and access to clear information are important parts of this process. They can help patients better understand their condition and the available treatment options. This can also support their involvement in medical decisions. Previous research among patients with chronic diseases has shown that communication and participation in shared decision-making are associated with higher levels of patient satisfaction [62]. These aspects are particularly relevant to the present study, which examines communication, patient involvement, participation in medical decision-making, and satisfaction in internal medicine. Further research is still needed to support the broader implementation of patient-centered care within healthcare systems [82].

Regarding the contribution of the study, several established dimensions of patient-centered care were integrated into a single empirical model within the context of Romanian internal medicine. It also examined the relationship between patient participation in medical decision-making and patient satisfaction. By bringing these factors together, the study provides an integrated perspective on patient participation and satisfaction among patients with chronic conditions receiving internal medicine care.

The results showed that doctor–patient communication was positively associated with participation in the decision-making process. This finding is consistent with the studies conducted by Land et al. [59], Siebinga et al. [60], and Bomhof-Roordink et al. [61]. In their studies, the authors highlighted the role of communication and information exchange in involving patients in decision-making. Furthermore, this study showed that trust in doctors was positively associated with patient participation in medical decision-making. This finding is closely related to the results of previous studies by Kraetschmer et al. [63] and Tian et al. [62]. The results are consistent with those previously obtained by Shen et al. [66] and Holden et al. [67]. In these two studies, the authors showed the importance of health literacy for active patient involvement. Another important result of this study concerns the positive association between patients’ involvement in their own care and their participation in medical decision-making. The findings are consistent with those reported by Smith et al. [69] and Poon et al. [70]. The quality of the information provided by the doctor was also positively associated with participation in medical decision-making. This finding was previously supported by Bomhof-Roordink et al. [61] and Richter et al. [68].

The strongest relationship identified in the model was between patient participation in medical decision-making and patient satisfaction. This relationship was also tested previously in studies conducted by Carlsen and Aakvik [75], Shay and Lafata [76], and Siebinga et al. [60]. This finding highlights the importance of considering patients’ preferences and experiences when discussing treatment options in internal medicine. However, this strong association should be interpreted with caution. Both patient participation in medical decision-making and patient satisfaction were measured using single items. These measures may not capture all dimensions of the two concepts. Therefore, the result reflects an association between the patients’ overall perceptions rather than a relationship between all aspects of participation and satisfaction.

Measuring patient-centered care is important for assessing healthcare quality. Previous systematic reviews have examined the validity and reliability of tools such as P-CAT [83] and CarerQol [84]. CarerQol focuses on caregivers’ quality of life rather than patients’ experiences. The present study focused on communication, information, patient involvement, and satisfaction. Future studies could use validated tools to further assess these aspects of care.

Patient-centered care also depends on healthcare professionals and their working conditions. Previous research has examined sense of coherence, work stress, and well-being among care professionals [85]. Studies involving professional caregivers working with people with mental illness have also addressed person-centered care [86]. These aspects were not examined in the present study and should be considered in future research.

Regarding the limitations of the research, a first limitation is related to the sampling method that was used. Snowball sampling was used, so the sample may not represent all patients with chronic conditions. The results mainly apply to the patients in this study and may be relevant to similar healthcare settings; they cannot be generalized to all patients in Romania or other countries. Future studies should include larger samples from different countries and healthcare settings. The sample was also relatively small (n = 176), which further limits the generalizability of the findings. Another limitation concerns the respondents included in the study population. The study included people who had attended medical consultations during the previous 12 months. Due to this period, some patients may not clearly remember their entire experience. Another limitation of the research concerns the variables considered in the conceptual model. The literature also presents other variables that were not included in this study. In addition, PPMD and patient satisfaction were each measured using a single-item scale, which may not fully reflect the complexity of these constructs. Both concepts are complex and may include several dimensions that cannot be fully captured by one question. As a result, the measures may not reflect all aspects of patients’ participation or satisfaction. In addition, the internal consistency of these two variables could not be assessed. This limitation should be considered when interpreting the structural relationships, particularly the association between patient participation and satisfaction. The findings reflect patients’ overall perceptions of these concepts and should not be interpreted as evidence of relationships between all their underlying dimensions. Future studies should use validated multi-item scales to provide a more comprehensive assessment of both constructs. Another limitation is the potential influence of common method bias. All variables were measured using the same self-reported questionnaire at a single point in time. Common method variance was not formally assessed. Therefore, its possible influence on the observed relationships cannot be ruled out, particularly the strong association between patient participation in medical decision-making and patient satisfaction (β = 0.75). Future studies could use data collected at different time points or from different sources to reduce this risk. Another limitation is the cross-sectional design of the study. As all data were collected at a single point in time, the relationships identified in the model should be interpreted as statistical associations rather than causal effects. Therefore, the results do not establish whether greater patient participation leads to higher satisfaction.

Based on the limitations of the research mentioned above, future studies could bring additional value by extending the proposed conceptual model. Thus, to confirm or not the results obtained in this study, future research should be conducted on larger samples. Moreover, other variables that were not included in the current study should be considered in the PLS-SEM model. Some recommended variables are doctor empathy, accessibility of medical services, and previous medical experiences. Future studies should also examine whether there are significant differences between different groups of patients, depending on their chronic conditions, age, sex, or level of education. Comparisons could also be made between patients who received consultations in private healthcare settings and those who received consultations in public healthcare settings. Future studies could also make it possible to follow patient behavior over time. This could show how the decision-making process evolves among patients, as well as their level of satisfaction. Moreover, the research could be extended to other countries, which would make it possible to compare the results. These future research directions could contribute to a better understanding of patient participation in medical decision-making and its role in improving patient-centered care.

## 5. Conclusions

Patient-centered care is considered one of the most important components of internal medicine, particularly for people with chronic conditions. Previous studies have highlighted the importance of actively involving patients in their own care. They have also emphasized the need to consider patients’ needs, values, and preferences. Previous findings have shown that shared decision-making is an important component of patient-centered care. This process involves collaboration between the patient and the doctor when treatment decisions are made. This approach emphasizes clear information, patient involvement, and attention to patients’ experiences with healthcare services. The present study provides a new applied perspective on patient-centered care among people with chronic conditions who received internal medicine services. The aim of the study was to identify the main factors associated with patient participation in medical decision-making and to examine the relationship between patient participation and patient satisfaction. The conceptual model developed in this study was intended to examine how these factors are associated with patient participation in the decision-making process. The findings of this study can contribute both to the existing literature and to medical practice. From a theoretical perspective, a new conceptual model was developed in the field of internal medicine. It illustrates the relationships between several patient-centered care factors, patient participation in medical decision-making, and patient satisfaction. From a practical perspective, the study highlights the main aspects that doctors working in this field should consider in order to improve their relationships with patients. The study also highlights the importance of moving from a disease-focused approach to one in which the patient is an active participant in decisions about their own care. In this context, supporting patient participation in medical decision-making may be an important step toward improving patient experience and developing healthcare services that are better aligned with patients’ needs. The findings show associations between the variables examined. Due to the cross-sectional design, causal relationships cannot be established.

## Figures and Tables

**Table 1:** Reliability and convergent validity of the constructs.

Construct	Cronbach’s α	Composite Reliability	AVE
Doctor–Patient Communication (DPC)	0.860	0.905	0.704
Trust in the Doctor (TD)	0.854	0.901	0.696
Ability to Understand Medical Information (AUMI)	0.829	0.887	0.662
Patient Involvement in Their Own Care (PIC)	0.858	0.904	0.702
Quality of Information Provided (QIP)	0.845	0.896	0.684

**Table 2:** Inner VIF values of the predictor constructs.

Endogenous Construct	Predictor	Inner VIF
PPMD	DPC	1.063
PPMD	TD	1.065
PPMD	AUMI	1.115
PPMD	PIC	1.058
PPMD	QIP	1.146
PS	PPMD	1.000

**Table 3:** Discriminant validity based on the Fornell–Larcker criterion.

Construct	DPC	TD	AUMI	PIC	QIP	PPMD	PS
DPC	0.839	0.133	0.183	0.117	0.138	0.505	0.355
TD	0.133	0.834	0.110	0.100	0.175	0.399	0.399
AUMI	0.183	0.110	0.814	0.115	0.261	0.514	0.358
PIC	0.117	0.100	0.115	0.838	0.199	0.473	0.351
QIP	0.138	0.175	0.261	0.199	0.827	0.441	0.250
PPMD	0.505	0.399	0.514	0.473	0.441	1.000	0.740
PS	0.355	0.399	0.358	0.351	0.250	0.740	1.000

**Table 4:** Discriminant validity assessment using the heterotrait–monotrait ratio (HTMT).

Construct	DPC	TD	AUMI	PIC	QIP
DPC	—				
TD	0.159	—			
AUMI	0.218	0.131	—		
PIC	0.137	0.118	0.135	—	
QIP	0.164	0.206	0.317	0.231	—

Note: The dash (—) indicates that the HTMT ratio is not applicable when a construct is compared with itself.

**Table 5:** KMO measure and Bartlett’s test of sphericity.

Indicator	Value
Kaiser–Meyer–Olkin (KMO) Measure of Sampling Adequacy	0.778
Bartlett’s Test of Sphericity—Approx. Chi-Square	1603.449
df	190
*p*-value	<0.001

**Table 6:** Results of hypothesis testing, effect sizes, and approximate 95% confidence intervals.

Hypothesis	Relationship	β	SE	Approx. 95% CI	Effect Size	*p*-Value	Decision
H1	DPC-PPMD	0.35	0.043	[0.266, 0.434]	0.176	<0.01	Supported
H2	TD-PPMD	0.24	0.044	[0.154, 0.326]	0.096	<0.01	Supported
H3	AUMI-PPMD	0.33	0.044	[0.244, 0.416]	0.169	<0.01	Supported
H4	PIC-PPMD	0.34	0.048	[0.246, 0.434]	0.164	<0.01	Supported
H5	QIP-PPMD	0.19	0.047	[0.098, 0.282]	0.086	<0.01	Supported
H6	PPMD-PS	0.75	0.039	[0.674, 0.826]	0.562	<0.01	Supported

β = standardized path coefficient; SE = standard error; CI = confidence interval. Effect sizes are reported as provided by WarpPLS 8.0. Approximate 95% confidence intervals were calculated as β ± 1.96 × SE using standard errors obtained from bootstrapping with 999 resamples.

**Table 7:** Coefficients of determination (R^2^) and predictive relevance (Q^2^).

Endogenous Construct	R^2^	Q^2^
Patient Participation in Medical Decision-Making (PPMD)	0.693	0.696
Patient Satisfaction (PS)	0.562	0.563

**Table 8:** Model fit and quality indices.

Indicator	Obtained Value	Validation Criterion
Average path coefficient (APC)	0.366, *p* < 0.001	*p* < 0.05
Average R-squared (ARS)	0.627, *p* < 0.001	*p* < 0.05
Average adjusted R-squared (AARS)	0.621, *p* < 0.001	*p* < 0.05
Average block VIF (AVIF)	1.089	Acceptable ≤ 5; ideally ≤ 3.3
Average full collinearity VIF (AFVIF)	1.954	Acceptable ≤ 5; ideally ≤ 3.3
Tenenhaus GoF (GoF)	0.699	Small ≥ 0.10; medium ≥ 0.25; large ≥ 0.36
Simpson’s paradox ratio (SPR)	1.000	Acceptable ≥ 0.70; ideally = 1
R-squared contribution ratio (RSCR)	1.000	Acceptable ≥ 0.90; ideally = 1
Statistical suppression ratio (SSR)	1.000	Acceptable ≥ 0.70
Nonlinear bivariate causality direction ratio (NLBCDR)	1.000	Acceptable ≥ 0.70

## Data Availability

The data presented in this study are available on request from the corresponding authors because the study data were collected within an institutional setting and are subject to ethical and data protection restrictions.
